# Rate and Outcomes of Second Visits to the ED Among Patients Discharged With Non-specific Abdominal Pain in a Tertiary Hospital in Saudi Arabia

**DOI:** 10.7759/cureus.93464

**Published:** 2025-09-29

**Authors:** Munira Alkhashan, Rakan Alsinaideh, Salman Alkhodairy, Mohammed Alqadhibi, Abdulaziz Alanzan

**Affiliations:** 1 Emergency Medicine, King Abdulaziz Medical City, Riyadh, SAU; 2 General Surgery, King Fahd Specialist Hospital, Qassim, SAU; 3 Anesthesiology, Prince Sultan Military Medical City, Riyadh, SAU

**Keywords:** appendicitis, cholecystitis, emergency department revisits, er, nonspecific abdominal pain

## Abstract

Background

It is well documented in the literature that the most common diagnosis for acute abdomen is nonspecific abdominal pain (NSAP). The context of NSAP in the literature is either (a) ranking it among other causes of acute abdomen or (b) addressing that some patients revisited the ED with a different diagnosis after being initially labeled with NSAP. These two contexts create a gap, as no article has discussed the characteristics of patients mislabeled with NSAP in KSA. In other words, what might be the risk factors making their presentation nonspecific? In this article, we discuss the characteristics of patients labeled with NSAP who revisited the ED within three days and were subsequently diagnosed with a different condition, if found.

Methodology

The study was a retrospective cohort study. Data were taken from case files at King Abdulaziz Medical City (KAMC), a tertiary hospital in Riyadh, Saudi Arabia, using the Best-Care system. All adults who attended the ED and were discharged with the diagnosis of NSAP (ICD code R10.4) between 2015 and 2023 were included. Patients were then tracked for 3 days, which is the cutoff commonly used in ED research. Statistical analysis was performed using R 4.3. Categorical variables were summarized using counts and percentages, and continuous variables (normal and non-normal) using mean ± SD and median/IQR. The chi-square test was used to assess associations, and logistic regression analysis was used to predict revisits. Only statistically significant variables were included, and hypothesis testing was performed at a 5% level of significance.

Results

A total of 932 patients were discharged from the ED with NSAP. Out of these, 69 (7.4%) revisited the ED within 3 days and received a different diagnosis. The study found significant age differences between the two groups: the median age was 40 years (IQR: 27-58) in non-revisited patients and 29 years (IQR: 21-42) in re-visited patients. A higher proportion of re-visited patients had a BMI over 25 (64.1% vs. 40.6%, p < 0.001). Surgical history also showed a significant difference, with 39.1% of re-visited patients having a history of abdominal surgery compared to 18.2% of non-revisited patients (p < 0.001). Acute cholecystitis was the most common diagnosis during the second visit, accounting for 32.3% of revisits, followed by appendicitis. Re-visited patients also showed a higher prevalence of hypothyroidism (13.4% vs. 1.27%) and a higher incidence of type 2 diabetes (8.96% vs. 1.27%).

Conclusion

This retrospective cohort study revealed several significant findings regarding factors associated with ED revisits following an initial discharge with a diagnosis of NSAP. The most common diagnosis identified during the second visit was acute cholecystitis.

## Introduction

Acute abdominal pain is defined as “abdominal pain of nontraumatic origin with a maximum duration of 5 days” [1]. It is a common cause for people to seek medical attention in the ED [2]. Causes of acute abdominal pain vary from emergent conditions requiring immediate intervention to less serious causes [3]. Special diagnostic challenges are encountered in some patients, as they exhibit ambiguous and nonspecific symptoms, as well as unusual manifestations, leading to extensive and time-consuming investigations [4,5]. Consequently, even skilled and experienced doctors may find it difficult to diagnose the cause of acute abdominal pain [6]. Thus, in many cases, the etiology of acute abdominal pain cannot be determined, and patients may be diagnosed with nonspecific abdominal pain (NSAP) and discharged from the ED.

Acute NSAP is defined as “acute abdominal pain less than one week in duration, for which there is no diagnosis despite investigations and which comprises a spectrum of undiagnosed conditions, both somatic and functional,” and remains a diagnosis of exclusion [7]. Despite the availability of advanced diagnostic techniques, a significant proportion of patients (around 25% in ED discharges and 35-41% in admissions) are diagnosed with NSAP [8-10]. This may delay the diagnosis of a specific condition rather than NSAP itself. Therefore, evaluation of abdominal symptoms is difficult and highly challenging [11,12]. Considering the outcomes of delayed diagnosis and the risk of readmission with other conditions, it has been reported that the longer it takes for patients to receive a diagnosis and treatment, the greater the likelihood of negative outcomes such as death, morbidity, and psychological illness [13,14].

The primary objective of this study was to determine the 72-hour revisit rate among adult patients discharged with a diagnosis of NSAP at a tertiary hospital in Saudi Arabia. Secondary objectives were to (1) identify patient characteristics and risk factors associated with revisit and (2) describe the diagnoses made on second visits.

To our knowledge, there is no literature discussing the rate of NSAP in Riyadh, Saudi Arabia, and little literature worldwide addressing ED revisits after a diagnosis of NSAP. This study, therefore, aims to provide a valuable contribution to the Saudi medical literature.

## Materials and methods

This study was designed as a retrospective cohort chart review conducted at King Abdulaziz Medical City (KAMC), Riyadh, Saudi Arabia, a tertiary care level-1 trauma center. The study period spanned from January 2015 to December 2023.

Inclusion and exclusion criteria

We included all adult patients aged 18 years and above who presented to the ED and were discharged with a diagnosis of NSAP, coded under the International Classification of Diseases (ICD-10) code R10.4.

Exclusion criteria were patients younger than 18 years, patients with trauma-related abdominal pain, pregnant women, those with incomplete or missing medical records, and patients who received a confirmed diagnosis other than NSAP during the index visit.

Outcome definition

A revisit was defined as a return presentation to the same ED within 72 hours of discharge. A “different diagnosis” was defined as a diagnosis at the second visit that differed from the initial NSAP (ICD-10 R10.4) discharge code. Diagnoses were determined through manual chart review of the electronic medical records and adjudicated by emergency physicians. Revisits to other hospitals were not captured, which may underestimate the true revisit rate.

Data collection 

Data were extracted from the hospital’s electronic medical record system (Best-Care) by trained medical data abstractors using a standardized Excel form. To ensure accuracy, a random 10% sample was cross-checked by senior clinicians. Any discrepancies were resolved by consensus. Variables collected included demographic information (age, sex, BMI, blood type), vital signs (temperature, heart rate), laboratory parameters (WBC count), clinical characteristics (pain type, pain radiation, associated symptoms), surgical history, imaging performed during the index visit, comorbidities, and subsequent diagnoses at the revisit.

Handling of missing data 

We recorded the percentage of missing values for key variables. Missing data were managed using complete-case analysis without imputation.

Symptom coding 

Symptoms were categorized based on clinical documentation in the EMR. Composite symptom categories (e.g., “nausea and anorexia”) were created when both symptoms were recorded concurrently. Recoding rules were predefined and applied consistently across the dataset.

Statistical analysis 

All analyses were performed using R software (version 4.3). Categorical variables were summarized as counts and percentages, while continuous variables were expressed as mean ± SD for normally distributed data or as median with IQR for non-normal data. Associations between categorical variables were tested using the chi-square test. Logistic regression analysis was conducted to identify factors associated with ED revisits, with a backward elimination approach used for variable selection. Model performance was assessed using the AUC and calibration plots. Given the limited number of events (n = 69 revisits), the possibility of model overfitting was acknowledged. A two-tailed p-value < 0.05 was considered statistically significant.

## Results

The study encompassed a cohort of 932 patients, with 69 (7.4%) re-visiting the ER within three days. Analysis revealed significant differences in age distribution between the two groups, with a median age of 40 years (IQR: 27-58) in the non-revisited group compared to a younger median age of 29 years (IQR: 21-42) in re-visited patients (p < 0.001). Gender distribution did not significantly differ between the groups (p = 0.860). BMI categories indicated significant differences; two-thirds of patients who were not re-visited had a BMI of less than 25 (64.1%), compared to 40.6% of those who were re-visited (p < 0.001). Blood type distributions did not significantly correlate with re-visit (p = 0.267) (Table 1).

**Table 1:** Descriptive statistics for the study sample stratified by re-visit.

Parameter	All (N=932)	No Re-visit (N=863)	Re-visit (N=69)	p-value
Age	39.0 (26.0; 56.0)	40.0 (27.0; 58.0)	29.0 (21.0; 42.0)	<0.001
Gender				
Female	416 (44.6%)	384 (44.5%)	32 (46.4%)	
Male	516 (55.4%)	479 (55.5%)	37 (53.6%)	
BMI				
< 25	581 (62.3%)	553 (64.1%)	28 (40.6%)	<0.001
≥ 25	351 (37.7%)	310 (35.9%)	41 (59.4%)	
Blood type				
A+	41 (4.40%)	38 (4.40%)	3 (4.35%)	0.267
AB-	2 (0.21%)	2 (0.23%)	0 (0.00%)	
AB+	6 (0.64%)	5 (0.58%)	1 (1.45%)	
B-	9 (0.97%)	8 (0.93%)	1 (1.45%)	
B+	37 (3.97%)	33 (3.82%)	4 (5.80%)	
O-	16 (1.72%)	13 (1.51%)	3 (4.35%)	
O+	821 (88.1%)	764 (88.5%)	57 (82.6%)	
Initial heart rate				
< 60	37 (3.97%)	34 (3.94%)	3 (4.35%)	0.142
60-100	387 (41.5%)	351 (40.7%)	36 (52.2%)	
> 100	508 (54.5%)	478 (55.4%)	30 (43.5%)	
Frequency of ED visits per year	2.00 (2.00; 4.00)	2.00 (2.00; 3.00)	4.00 (3.00; 4.00)	<0.001
Temperature				
Hyperpyrexia	3 (0.32%)	3 (0.35%)	0 (0.00%)	0.185
Hypothermia	3 (0.32%)	3 (0.35%)	0 (0.00%)	
> 37.5 °C	205 (22.0%)	183 (21.2%)	22 (31.9%)	
Normal	721 (77.4%)	674 (78.1%)	47 (68.1%)	
Initial WBC count				
Leukocytosis	105 (11.3%)	82 (9.50%)	23 (33.3%)	<0.001
Normal	827 (88.7%)	781 (90.5%)	46 (66.7%)	
Radiation of pain				
Diffuse	3 (0.32%)	1 (0.12%)	2 (2.94%)	0.005
Fixed	76 (8.16%)	70 (8.11%)	6 (8.82%)	
None	754 (81.0%)	707 (81.9%)	47 (69.1%)	
To back	37 (3.97%)	31 (3.59%)	6 (8.82%)	
To chest	28 (3.01%)	26 (3.01%)	2 (2.94%)	
To shoulder	33 (3.55%)	28 (3.25%)	5 (7.35%)	
Type of pain				0.001
Colicky	75 (8.06%)	64 (7.42%)	11 (16.4%)	0.018
Diffuse	155 (16.7%)	140 (16.2%)	15 (22.4%)	0.257
Dull	60 (6.45%)	56 (6.49%)	4 (5.97%)	0.999
Migrating	4 (0.43%)	3 (0.35%)	1 (1.49%)	0.259
Non-specific	606 (65.2%)	576 (66.7%)	30 (44.8%)	<0.001
Sharp	30 (3.23%)	24 (2.78%)	6 (8.96%)	0.017
Previous surgical history				
No	495 (53.1%)	471 (54.6%)	24 (34.8%)	<0.001
Abdominal	184 (19.7%)	157 (18.2%)	27 (39.1%)	
Non-abdominal	253 (27.1%)	235 (27.2%)	18 (26.1%)	
Diagnostic workup (first visit)				
CT	297 (31.9%)	270 (31.3%)	27 (39.7%)	0.001
US	325 (34.9%)	293 (34.0%)	32 (47.1%)	
X-ray	309 (33.2%)	300 (34.8%)	9 (13.2%)	
Disposition of the patient (second visit)				
Admission	-	-	56 (81.2%)	
Discharged	-	-	13 (18.8%)	

The initial heart rate was not significantly different between groups (p = 0.142). The frequency of ED visits per year was significantly higher in re-visited patients (median = 4 visits, IQR: 3-4), compared to a median of 2 visits (IQR: 2-3) in those who were not re-visited (p < 0.001). The prevalence of leukocytosis was higher in re-visited patients compared to those who were not re-visited (33.3% vs. 9.5%, p < 0.001). The distribution of pain types was significantly different between groups (p = 0.001). Temperature was not significantly different between groups.

Surgical history was significantly different between groups, with 39.1% of re-visited patients having a history of abdominal surgery compared to 18.2% of non-revisited patients (p < 0.001). Diagnostic procedures during the first visit, such as CT and US, were more common in re-visited patients (p = 0.001).

**Figure 1:**
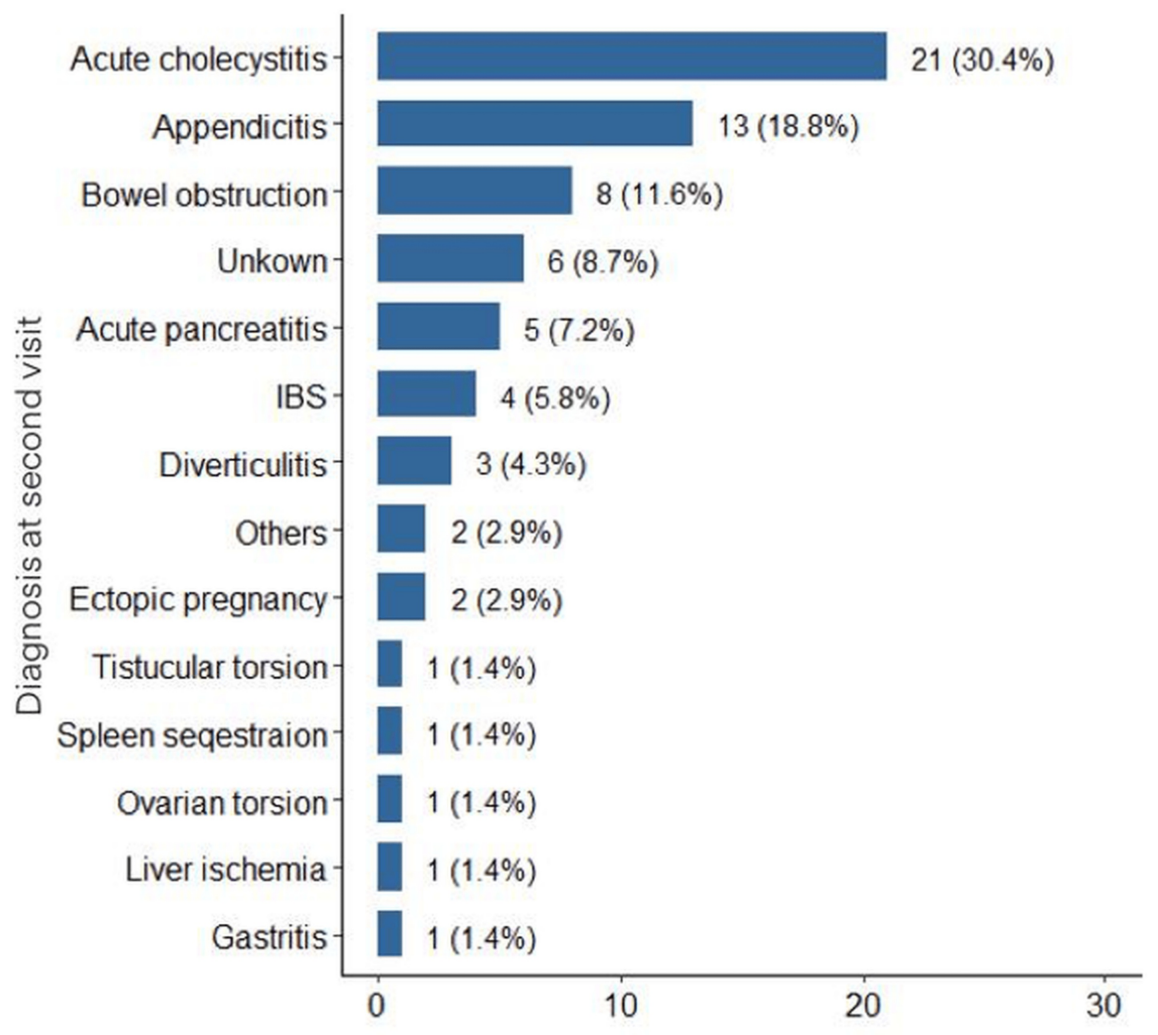
Diagnosis at second visit. IBS: Irritable Bowel Syndrome.

The most frequent diagnosis during the second visit was acute cholecystitis, accounting for 30.4% (n = 21) of the total re-visits, followed by appendicitis, which comprised 18.8% (n = 13) of the cases. Bowel obstruction was the third most common condition leading to re-visit, representing 11.6% (n = 8). The diagnosis was unknown in 8.7% of the re-visited cases. The remaining cases included a variety of conditions such as acute pancreatitis, other surgical pathologies, and gastrointestinal issues, each constituting a smaller fraction of the total re-visits (Figure 1).

**Figure 2:**
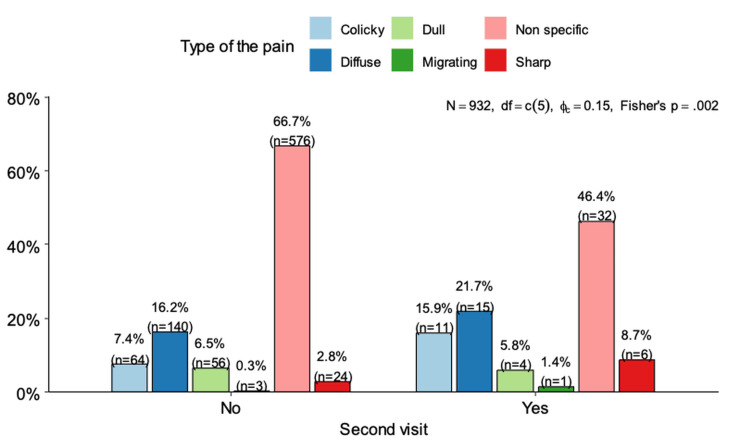
Association between type of pain and re-visit.

Figure 2 illustrates the relationship between the type of pain patients reported and their subsequent hospital re-visit. Those who were not re-visited predominantly reported non-specific pain, constituting 66.7% (n = 576). Other pain descriptions in the non-revisited cohort included colicky pain (15.9%, n = 140), dull pain (7.4%, n = 64), diffuse pain (6.5%, n = 56), sharp pain (2.8%, n = 24), and migrating pain (0.3%, n = 3). Among the re-visited patients, non-specific pain was still common at 46.4% (n = 30), but there was a noticeably higher incidence of colicky pain at 15.9% (n = 11), suggesting a stronger association with re-visit. Diffuse pain was reported by 21.7% (n = 15) and dull pain by 5.8% (n = 4) in the re-visited group (Figure 2). The distribution of comorbidities between re-visited and non-revisited patients is summarized in Table 2.

**Table 2:** Association between re-visit and comorbidities. Note: Data were summarized using counts and percentages. Analysis was performed using the Chi-square test of independence. SCD: Sickle Cell Disease; DM: Diabetes Mellitus; CKD: Chronic kidney disease

Comorbidity	No re-visit (N=863)	Re-visit (N=69)	p-value
Asthma	42 (4.87%)	7 (10.4%)	0.078
Hypothyroidism	11 (1.27%)	9 (13.4%)	<0.001
DM2 (Type 2 DM)	230 (26.7%)	22 (32.8%)	0.34
Hypertension (HTN)	201 (23.3%)	19 (28.4%)	0.429
CKD	9 (1.04%)	0 (0.00%)	1
DM1 (Type 1 DM)	11 (1.27%)	6 (8.96%)	0.001
Heart failure	6 (0.70%)	0 (0.00%)	1
Hyperthyroidism	14 (1.62%)	1 (1.49%)	1
Stroke	10 (1.16%)	1 (1.49%)	0.563
Medically free	449 (52.0%)	18 (26.9%)	<0.001
Osteoarthritis	2 (0.23%)	0 (0.00%)	1
SCD	0 (0.00%)	1 (1.49%)	0.072

The prevalence of asthma and hypothyroidism was higher in the re-visited group, with asthma present in 10.4% compared to 4.87% in the non-revisited group (p = 0.078), suggesting a trend towards significance. Hypothyroidism was significantly more common among re-visited patients, affecting 13.4% compared to only 1.27% of those not re-visited (p < 0.001). Diabetes mellitus type 2 (DM2) was observed in 26.7% of the non-revisited group and 32.8% of the re-visited group (p = 0.340). Hypertension (HTN) was present in 23.3% of the non-revisited group and 28.4% of the re-visited group (p = 0.429). Chronic kidney disease (CKD), heart failure, hyperthyroidism, stroke, and osteoarthritis were present in a small percentage of both groups, with no significant differences observed. The absence of comorbidities was significantly more common in the non-revisited group, at 52% compared to only 26.9% in the re-visited group (p < 0.001) (Table 3).

**Table 3:** Association between symptoms and re-visit. Note: Data were summarized using counts and percentages. Analysis was performed using the Chi-square test of independence.

Symptom	No re-visit (N=863)	Re-visit (N=69)	p-value
Anorexia	57 (6.64%)	1 (1.45%)	0.117
Ascites	1 (0.12%)	0 (0.00%)	1
Back pain	39 (4.55%)	1 (1.45%)	0.355
Constipation	69 (8.04%)	14 (20.3%)	0.001
Cough	1 (0.12%)	0 (0.00%)	1
Diaphoresis	0 (0.00%)	1 (1.45%)	0.074
Diarrhea	41 (4.78%)	4 (5.80%)	0.571
Dyspnea	7 (0.82%)	0 (0.00%)	1
Dysuria	24 (2.80%)	1 (1.45%)	1
Fatigue	96 (11.2%)	3 (4.35%)	0.117
Fever	42 (4.90%)	1 (1.45%)	0.364
Fever and anorexia	11 (1.28%)	3 (4.35%)	0.079
Fever and constipation	0 (0.00%)	2 (2.90%)	0.005
Fever and diarrhea	22 (2.56%)	1 (1.45%)	1
Fever and nausea	6 (0.70%)	0 (0.00%)	1
Fever and vomiting	18 (2.10%)	0 (0.00%)	0.389
Fever, nausea, vomiting	56 (6.53%)	8 (11.6%)	0.133
Gas and bloating	56 (6.53%)	3 (4.35%)	0.614
Headache	84 (9.79%)	3 (4.35%)	0.202
Nausea	12 (1.40%)	2 (2.90%)	0.28
Nausea and anorexia	0 (0.00%)	9 (13.0%)	<0.001
Nausea and vomiting	64 (7.46%)	10 (14.5%)	0.065
None	123 (14.3%)	2 (2.90%)	0.013
Polyuria and fatigue	7 (0.82%)	0 (0.00%)	1
Vomiting	20 (2.33%)	0 (0.00%)	0.391
Weight loss	2 (0.23%)	0 (0.00%)	1

Significant differences were observed in constipation and in the combined symptoms of nausea and anorexia. Specifically, constipation was significantly more prevalent in the re-visited group, with 20.3% of patients experiencing this symptom compared to 8.04% in the non-revisited group (p = 0.001). Furthermore, the co-occurrence of nausea and anorexia was notably higher in the re-visited group, with 13.0% reporting these symptoms versus none in the non-revisited group, highlighting a significant association with re-visit (p < 0.001). Additionally, the presence of fever combined with constipation was statistically significant in the re-visited group, with 2.90% experiencing these symptoms (p = 0.005). Lastly, there was a significant decrease in patients reporting no symptoms in the re-visited group (2.90%) compared to the non-revisited group (14.3%, p = 0.013), suggesting that the absence of symptoms is less likely in those who re-visited (Table 4).

**Table 4:** Association between symptoms and re-visit after recoding Note: Data were summarized using counts and percentages. Analysis was performed using the Chi-square test of independence.

Symptom	No (N=863)	Yes (N=69)	p-value
Anorexia	68 (7.93%)	13 (18.8%)	0.004
Ascites	1 (0.12%)	0 (0.00%)	1
Back pain	39 (4.55%)	1 (1.45%)	0.355
Constipation	69 (8.04%)	16 (23.2%)	<0.001
Cough	1 (0.12%)	0 (0.00%)	1
Diaphoresis	0 (0.00%)	1 (1.45%)	0.074
Diarrhea	63 (7.34%)	5 (7.25%)	1
Dyspnea	7 (0.82%)	0 (0.00%)	1
Dysuria	24 (2.80%)	1 (1.45%)	1
Fatigue	103 (12.0%)	3 (4.35%)	0.084
Fever	155 (18.1%)	15 (21.7%)	0.551
Nausea	138 (16.1%)	29 (42.0%)	<0.001
Vomiting	158 (18.4%)	18 (26.1%)	0.16
Gas and bloating	56 (6.53%)	3 (4.35%)	0.614
Headache	84 (9.79%)	3 (4.35%)	0.202
None	123 (14.3%)	2 (2.90%)	0.013
Polyuria	7 (0.82%)	0 (0.00%)	1
Weight loss	2 (0.23%)	0 (0.00%)	1

With each additional year of age, the odds of re-visit decreased (OR = 0.95, 95% CI: 0.93-0.97, p < 0.001). Normal levels of WBC compared to leukocytosis were associated with about half the odds of re-visit, although this was marginally significant (OR = 0.49, 95% CI: 0.23-1.02, p = 0.056). The presence of nausea at presentation increased the odds of re-visit more than threefold (OR = 3.05, 95% CI: 1.49-6.23, p = 0.002). Patients with constipation had more than eight times the odds of re-visit (OR = 8.24, 95% CI: 3.61-18.82, p < 0.001). The presence of anorexia was also associated with a sixfold increase in the odds of re-visit (OR = 6.40, 95% CI: 2.65-15.48, p < 0.001). Those with no history of abdominal surgery had lower odds of re-visit compared to those with such a history (OR = 0.24, 95% CI: 0.12-0.51, p < 0.001), while previous non-abdominal surgery was not associated with re-visit (OR = 0.50, 95% CI: 0.23-1.11, p = 0.088).

Experiencing colicky pain increased the odds of re-visit (OR = 3.54, 95% CI: 1.46-8.58, p = 0.005). Hypothyroidism was also associated with higher odds of re-visit (OR = 3.24, 95% CI: 0.97-10.75, p = 0.055). Individuals with type 2 diabetes had significantly higher odds of re-visit (OR = 6.61, 95% CI: 1.81-24.13, p = 0.004). Overweight individuals had three times the odds of re-visit compared to those with a normal BMI (OR = 3.01, 95% CI: 1.56-5.78, p = 0.001). The model’s R² value of 0.362 indicated that these predictors explained approximately 36.2% of the variability in re-visit (Table 5).

**Table 5:** Logistic regression analysis of factors associated with re-visit. Model R² = 0.362.

Predictor	Odds Ratio (OR)	95% CI	p-value
Intercept	0.47	0.14-1.65	0.241
Age	0.95	0.93-0.97	<0.001
WBC: Normal vs. Leukocytosis	0.49	0.23-1.02	0.056
Nausea: Yes vs. No	3.05	1.49-6.23	0.002
Constipation: Yes vs. No	8.24	3.61-18.82	<0.001
Anorexia: Yes vs. No	6.4	2.65-15.48	<0.001
Previous surgery: No vs. Abdominal	0.24	0.12-0.51	<0.001
Previous surgery: Non-abdominal vs. Abdominal	0.5	0.23-1.11	0.088
Colicky pain: Yes vs. No	3.54	1.46-8.58	0.005
Hypothyroidism: Yes vs. No	3.24	0.97-10.75	0.055
Diabetes type 1: Yes vs. No	6.61	1.81-24.13	0.004
BMI: Overweight vs. Normal	3.01	1.56-5.78	0.001

## Discussion

This study evaluated revisit patterns among patients discharged from the ED with NSAP. We found that approximately 7% of patients returned within 72 hours, most commonly with acute cholecystitis and appendicitis. These findings highlight the clinical relevance of NSAP, a common yet diagnostically challenging presentation, and underscore the importance of appropriate discharge planning and follow-up.

Revisit rate and diagnoses

The 7% revisit rate observed in our study is comparable to international reports. Studies from the United States and Europe have documented revisit rates between 5% and 10% among NSAP patients [2,8-10]. Similar results have been reported in Asian cohorts, where appendicitis and biliary disease were among the leading causes of return visits [14]. Abdominal pain presentations also vary significantly between younger and older populations, as highlighted in previous studies of elderly patients [15,16]. The most frequent second-visit diagnoses in our cohort, appendicitis and cholecystitis, were consistent with international literature [17-19]. These consistencies suggest that the diagnostic uncertainty of NSAP is a global challenge rather than one limited to our setting.

Risk factors for revisit

Several factors were associated with higher revisit risk. Younger age emerged as a significant factor in our study, echoing findings that abdominal pain presentations differ between younger and older patients [4,5]. While older patients are often evaluated more extensively due to comorbidities, younger patients may be discharged earlier, potentially contributing to higher revisit rates.

Comorbidities also influenced outcomes. Type 1 diabetes was significantly associated with revisits, while Type 2 diabetes was not. Prior studies have shown that diabetes can complicate abdominal pain presentations due to atypical symptoms, making diagnosis more challenging [13]. Hypothyroidism, while significant in univariate analysis, was only borderline in regression and should be interpreted cautiously.

Other predictors included higher BMI, prior abdominal surgery, leukocytosis, and GI symptoms such as nausea, constipation, and anorexia. These associations are biologically plausible and align with previous studies highlighting the importance of systemic and gastrointestinal factors in missed diagnoses [6,7].

Clinical implications

Our findings, in line with international literature, suggest that certain subgroups of NSAP patients may require closer monitoring, additional diagnostic testing, or enhanced discharge instructions. In particular, younger patients, those with type 1 diabetes, and those presenting with gastrointestinal symptoms represent higher-risk groups. These implications are hypothesis-generating and warrant further investigation in prospective multicenter studies.

Limitations

This study has several important limitations that should be considered when interpreting the findings. First, it was conducted at a single tertiary care center, which may limit the generalizability of results to other settings with different patient populations, healthcare systems, or diagnostic resources. Second, only revisits to the same hospital were captured; patients who sought care at other facilities were not included. This may have led to an underestimation of the true revisit rate.

Third, the definition of a “different diagnosis” relied on electronic medical record documentation and coding, which introduces the potential for misclassification. Although manual chart review and physician adjudication were performed, diagnostic variability and coding errors cannot be completely excluded.

Fourth, data abstraction was retrospective and subject to limitations of the available records. Missing data were handled using complete-case analysis, which may introduce bias if missingness was not random. The proportion of missing values was not negligible for certain variables, and this should be taken into account when interpreting results.

Fifth, symptom recoding into composite categories (e.g., combining nausea and anorexia) may have introduced collinearity and reduced reproducibility. Although these categories were predefined, they remain subjective and could affect associations.

Sixth, the number of patients with revisits (n = 69) was relatively small compared with the number of variables considered in the regression model. This imbalance increases the risk of overfitting, despite the use of backward elimination, and limits the strength of inferences. Model performance metrics such as discrimination and calibration were included, but the small event count remains an inherent constraint.

Finally, the follow-up period was limited to 72 hours. While this window is clinically relevant for early return visits, it may not capture clinically significant revisits occurring after three days. Longer follow-up intervals, such as 5-7 days, may provide additional insights into delayed presentations of surgical abdominal pathology.

## Conclusions

In this retrospective single-center study, approximately 7% of patients discharged with NSAP revisited the ED within 72 hours, most commonly with acute cholecystitis or appendicitis. Younger age, type 1 diabetes, higher body mass index, prior abdominal surgery, leukocytosis, and GI symptoms such as nausea, constipation, and anorexia were associated with a higher risk of revisit. These findings highlight the importance of careful discharge planning and clear return precautions, particularly for patients in these higher-risk groups.

Our results are consistent with international studies reporting similar revisit rates and diagnostic patterns, underscoring that NSAP remains a global diagnostic challenge. While this study provides valuable region-specific data, its retrospective, single-center design and limited follow-up period necessitate cautious interpretation. Future multicenter, prospective studies with larger cohorts and extended follow-up are needed to validate these associations, enhance reproducibility, and guide evidence-based strategies for the management and safe discharge of NSAP patients.
